# Current perspectives and trends in acupuncture for sleep disorders: a bibliometric analysis

**DOI:** 10.3389/fpsyt.2024.1338455

**Published:** 2024-10-29

**Authors:** Yi Huang, Xihan Ying, Jieqi Zhang, Rong Hu, Yi Chen, Lei Wu, Bowen Chen, Kai Zhang, Kelin He, Ruijie Ma

**Affiliations:** ^1^ Key Laboratory of Acupuncture and Neurology of Zhejiang Province, The Third School of Clinical Medicine (School of Rehabilitation Medicine), Zhejiang Chinese Medical University, Hangzhou, China; ^2^ Department of Acupuncture, The Third Affiliated Hospital of Zhejiang Chinese Medical University, Hangzhou, China; ^3^ College of Acupuncture-Massage and Rehabilitation, Hunan University of Chinese medicine, Changsha, China

**Keywords:** acupuncture, sleep disorders, bibliometrics, visualization, insomnia, electroacupuncture

## Abstract

**Background:**

Limitations of conventional treatment methods for sleep disorders have driven the use and development of complementary and alternative therapies such as acupuncture. However, despite the surge in related studies, there is still a lack of visual analysis and detailed elaboration regarding the current status, international collaborations, and research hotspots of acupuncture for sleep disorders.

**Methods:**

We conducted a bibliometric analysis of publications on acupuncture for sleep disorders using the Web of Science Core Collection database from 2004 to 2023. We utilized the R package “bibliometrix” to count publications and citations, VOSviewer to create an inter-institutional referencing network, and CiteSpace to identify references and keywords with the highest citation bursts. Additionally, we employed a bibliometric online analysis platform designed for analyzing national partnerships.

**Results:**

A total of 432 pertinent papers were retrieved, with China being the most prolific contributor, accounting for 61.6% of the publications, followed by the United States and South Korea. Despite China’s high output, its average article citation rate and proportion of international collaborations were notably lower than those of the United States. Key research institutions such as the University of Hong Kong, Shanghai University of Traditional Chinese Medicine, Memorial Sloan Kettering Cancer Center, and Guangzhou University of Chinese Medicine have played significant roles in this field. Among authors, Ka-Fai Chung from the University of Hong Kong stood out as the most productive. In terms of journals, MEDICINE was the most active, while SLEEP was considered the most authoritative. The clinical effects of acupuncture for insomnia have garnered significant attention in recent years, with electroacupuncture emerging as the prevailing technique for addressing sleep disorders.

**Conclusion:**

This bibliometric study effectively outlines the basic framework of knowledge surrounding acupuncture for sleep disorders over the past two decades, covering publications, countries, institutions, authors, and sources. It highlights promising clinical effects and underlying mechanisms of acupuncture, particularly for secondary insomnia and specific sleep disorders like restless legs syndrome. Moving forward, the focus and challenge for future research lie in the development of standardized study protocols and harmonization of efficacy assessment metrics.

## 1 Introduction

Sleep disorders encompass abnormalities in the rhythm, quality, and behavior of sleep, frequently arising from disruptions in the intrinsic mechanisms of sleep-wakefulness or as a result of psychiatric or somatic disorders (1). They represent a prevalent concern among patients seeking medical consultations. To address the intricate clinical manifestations of sleep disorders, the third edition of the International Classification of Sleep Disorders edition 3 classifies them into seven groups: insomnia disorders, sleep-related breathing disorders, circadian rhythm sleep-wake disorders, central disorders of hypersomnolence, sleep-related movement disorders, parasomnias, and other sleep disorders (2). Sleep disorders have a pervasive impact on individuals across all age groups, with a trend towards younger demographics (3, 4). Incomplete statistics indicate that the occurrence of insomnia alone accounts for more than one-third of the global population, resulting in a substantial annual medical expenditure of approximately $100 billion (5, 6). In the short term, sleep disorders can contribute to the development of neurasthenia, memory impairment, and weakened immune function. Over the long term, these disorders significantly elevate the susceptibility to mood disorders such as anxiety and depression, as well as physical ailments including stroke, cancer, and heart disease, thereby posing a severe threat to both the physical and mental well-being of individuals (7).

Currently, the clinical treatment options for sleep disorders are very limited, primarily falling under the categories of pharmacologic and nonpharmacologic treatments. Pharmacotherapy is commonly used to treat patients with acute sleep disorders, but its long-term efficacy is uncertain and has been associated with withdrawal symptoms, cognitive deficits, and cardiac arrhythmias (8, 9). Nonpharmacological approaches encompass cognitive behavioral therapy, exercise, acupuncture, and transcranial magnetic stimulation (10). According to traditional Chinese medicine theory, meridians are the channels for qi and blood flow, and acupoints are specific locations on the meridians where qi and blood from organs enter and exit (11). Yin and yang are a group of relative concepts, and disease is often the outward manifestation of an imbalance of yin and yang. As one of the nonpharmacological treatments for sleep disorders, acupuncture enhances the body’s immunity to eradicate pathogenic elements, harmonizes yin and yang, and reinstates the organism to its optimal physiological state by stimulating specific acupoints on the body’s surface (12). Empirical investigations have substantiated the safety and efficacy of acupuncture in addressing diverse sleep disorders, including insomnia, restless leg syndrome, jet lag syndrome, and sleep-related bruxism (13–15). Basic research has confirmed that acupuncture restores circadian rhythms in sleep-deprived rodents and improves their performance on behavioral tests (16, 17). It may involve brain-derived neurotrophic factors, inflammatory cytokines, the hypothalamic-pituitary-adrenal axis, the gut microbiota, and other cellular events (18). With the growing global interest in sleep-related issues and the recent advocacy for acupuncture, there has been a noticeable surge in publications concerning the application of acupuncture in sleep disorder management. However, existing reviews have mainly focused on the clinical efficacy and specific techniques of acupuncture interventions for different sleep disorders while neglecting to comprehensively summarize and reflect on the history, research status, impact, and emerging themes of relevant publications in the field (19, 20).

Bibliometric analysis is a commonly used method of quantitative research on publications that summarizes the progress of a research topic and analyzes the contributions of authors, institutions, journals, and countries or regions through collecting, processing, and managing data from previous publications (21). In addition, bibliometric analysis can identify hotspots, emerging trends, and knowledge networks in a given field. In 2019 Wenya Pei et al. published a bibliometric analysis on acupuncture for insomnia (22). However, this study mainly quantitatively analyzed and ranked the countries, journals, authors, and references in this field based on the number of publications and citations. It did not elaborate on the international cooperation situation of countries and institutions, the development trend of authors, keywords, and citations, or a qualitative analysis of the research hotspots. There is a lack of bibliometric studies that provide an in-depth and comprehensive discussion of publications on acupuncture for the broad category of sleep disorders.

The Web of Science (WoS) is a comprehensive, multidisciplinary, core journal citation indexing database for accessing global scholarly information (23–25). With over 1.7 billion searchable citation records, it offers a rigorous screening process for academic journals, making it more internationally recognized compared to other databases such as Scopus and Google Scholar (24). Therefore, we conducted a systematic bibliometric analysis of publications on acupuncture for sleep disorders published in the Web of Science Core Collection (WoSCC) database between 2004 and 2023, including publication distribution, country, institution, source, authors, references, and keyword clustering. In addition, this paper provides an overview of research progress over the past 20 years and identifies research hotspots and trends in the field. In short, this is the first description of a bibliometric overview of research on acupuncture for sleep disorders. With this study, we aim to fill a gap in the existing literature and provide researchers, clinicians, and specialists with a comprehensive understanding of the field’s current outlook and potential future directions.

## 2 Materials and methods

### 2.1 Data collection and search strategy

**Figure 1:**
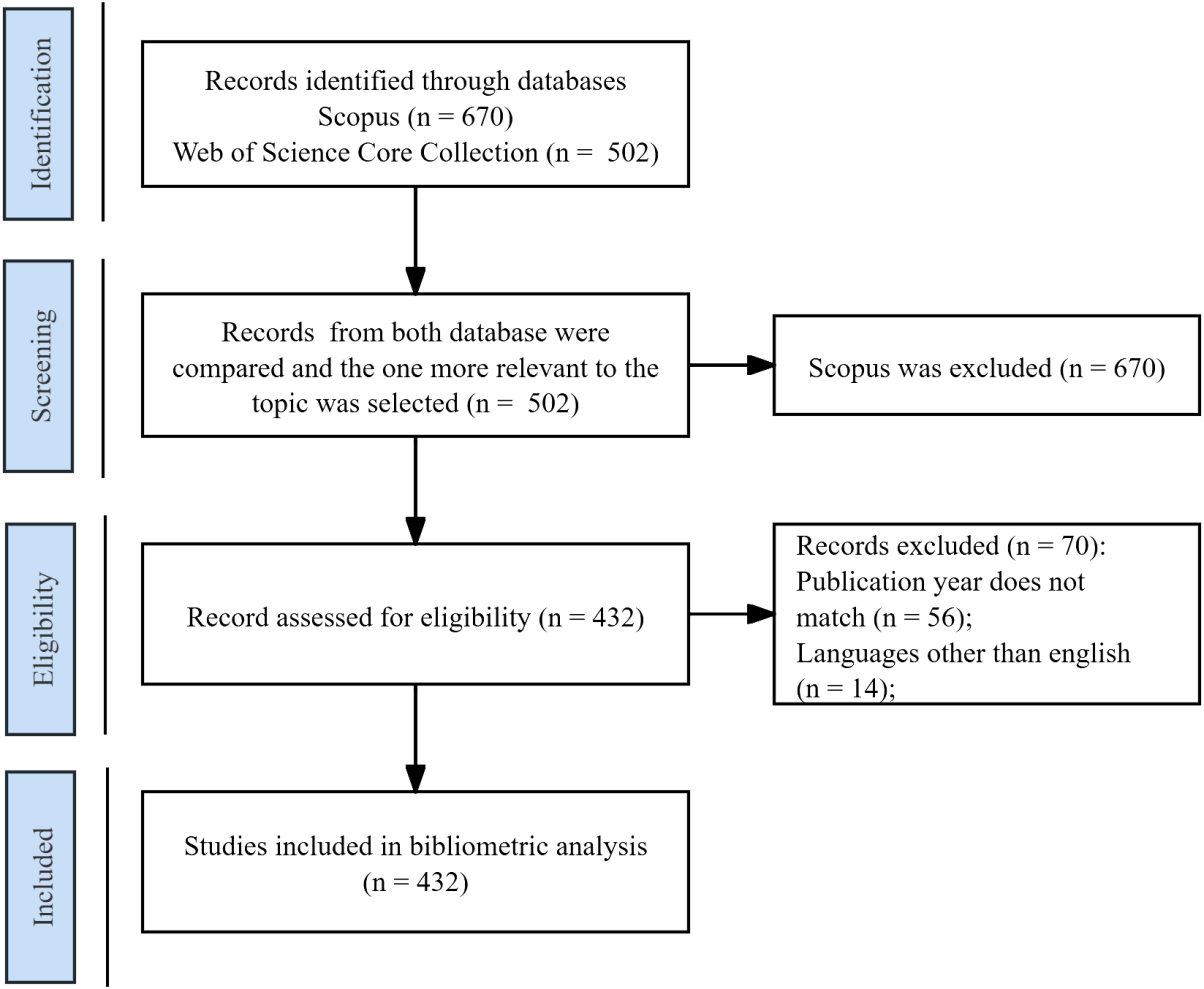
Study selection flowchart.

On October 6, 2024, we searched the WoSCC database for relevant publications. We used title searches to minimize the impact of non-directly related studies. Inclusion criteria were publication between January 1, 2004, and December 31, 2023, publication in English, and titles containing search terms related to “acupuncture” and “sleep disorders.” Exclusion criteria were the absence of two or more elements, such as title, abstract, source, publication date, references, authors, organization, duplication of studies, or inconsistent year of publication. The records of eligible publications were extracted and saved as “BibTex” and “plain text file” formats for further analysis. The specific search strategy and data collection process can be seen in **Table 1** and **Figure 1**.

**Table 1:** Search strategy.

Date run	Sunday, October 6, 2024	
Database	Web of Science Core Collection	
Search formula	AND	TI = (“acupuncture” OR “needling” OR “electroacupuncture” OR “electro-acupuncture” OR “warm needling” OR “warm acupuncture” OR “fire needle” OR “dry needling” OR “scalp acupuncture” OR “body acupuncture” OR “ear acupuncture” OR “auricular acupuncture” OR “abdominal acupuncture” OR “wristankle acupuncture” OR “manual acupuncture” OR “Moxibustion” OR “acupoint” OR “acupoint injection”)
		TI = (“sleep*” OR “insomnia” OR “sleep-related” OR “hypoventilation” OR “hypersomn*” OR “narcolepsy” OR “Kleine-Levin syndrome” OR “sleep-wake” OR “jet lag” OR “nightmare” OR “Parasomnia*” OR “confusional arousals” OR “exploding head syndrome” OR “restless legs syndrome” OR “periodic limb movement disorder”)
Publication year	Thursday, January 1,2004 - Sunday, December 31,2023	
Language	English	

### 2.2 Data analysis

For bibliometric analysis, five tools were used, namely Citespace (version 6.2.R4), R software (version 4.1.2), Bibliometric Online Analysis Platform (https://bibliometric.com/), VOSviewer (version 1.6.19) and Scimago Graphica (version 1.0. 44). CiteSpace, a citation visualization and analysis software based on the JAVA programming language, with Years per slice set to 1, G-index to 21, and pruning followed by visualization, was used to identify keywords with citation bursts and to perform clustering (25). The biometrics package in the R software ran and automatically invoked the default browser to open the biblioshiny web page to count the number of publications and citations by country, journal, and author (26). The bibliometrics online analysis platform analyzed national partnerships through World Wide Web services (27). The threshold was set to at least five publications, and a visualization network for institutional co-authorship analysis was constructed using VOSviewer, and combined with Scimago Graphica for beautification (28).

## 3 Results

### 3.1 Summary of publications

**Figure 2:**
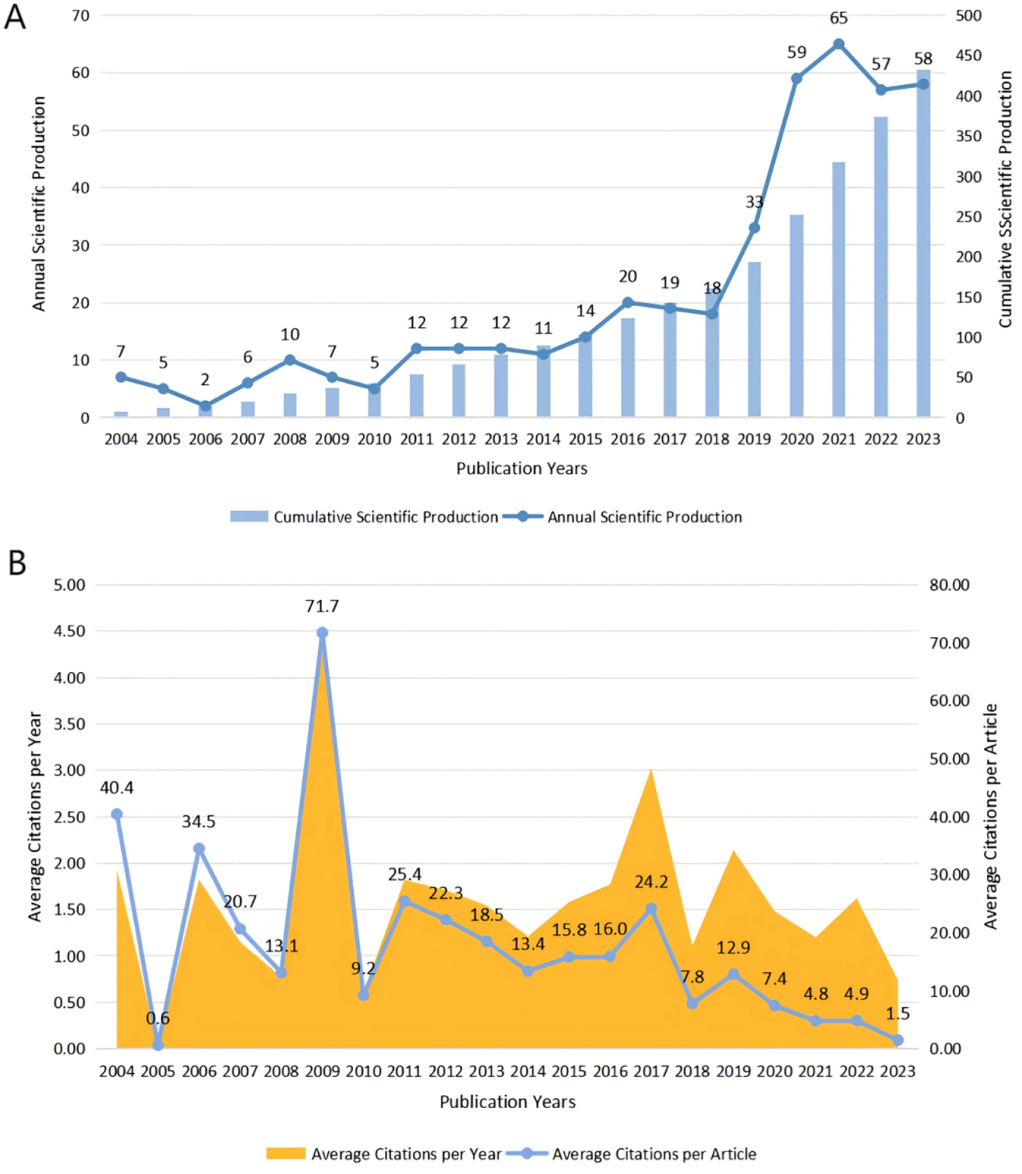
**(A)** Annual and cumulative scientific production; **(B)** average citations per article and per year.

Based on the eligibility criteria, a total of 432 English-language publications on acupuncture for sleep disorders published between 2004 and 2023 were included, with 251 regular articles (58.1%) and 97 review articles (22.45%). **Figure 2A** illustrates the quantity and trend of annual publications on acupuncture for sleep disorders. The average annual growth rate of pertinent publications over the previous two decades has been 11.77%. Among them, the yearly and cumulative number of publications from 2004 to 2018 increased gradually, with an average of 10.67 publications per year. It was not until 2015 that the cumulative number of publications exceeded 100, indicating that research on acupuncture for sleep disorders is still in its infancy. In the last five years, the number of publications has increased dramatically compared to the previous period, with the total number of articles not yet exceeding 500, potentially due to the limited prevalence of acupuncture techniques for treating sleep disorders.

The papers’ impact can be assessed by examining the average number of citations. **Figure 2B** shows that only seven articles on acupuncture for sleep disorders were published in 2009, However, each article received an average of 71.71 citations, and the average number of citations per year also peaked. This result indicates a high level of research output for that year. The average number of citations per article and per year has generally decreased since 2018. This decline may be attributed to the notable rise in relevant publications over the past five years compared to the previous period, while new publications are cited less frequently.

### 3.2 Country analysis

**Figure 3:**
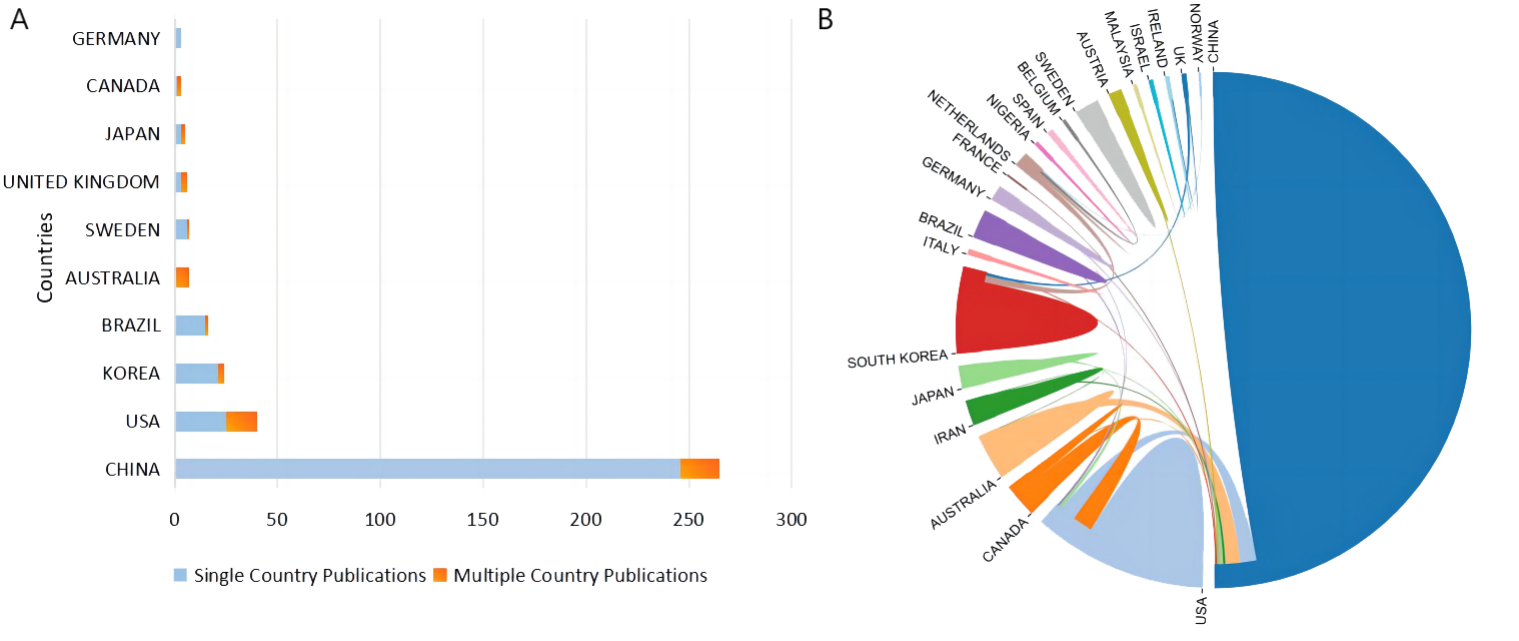
**(A)** National distribution of relevant authors; **(B)** National cooperation. The connecting lines indicate that the countries at either end of the line have cooperated; the thicker the line, the more frequent the cooperation. The area of each panel represents the percentage of relevant publications in each country; the larger the area, the greater the number of relevant publications.

A total of 27 countries have contributed to scientific research on acupuncture for sleep disorders. China emerged as the leading contributor, accounting for 61.6% of the publications, followed by the United States (U.S.), South Korea, and Brazil, while the rest of the countries had fewer than ten relevant publications (**Figure 3A**). It is worth noting that China published 266 papers, with transnational cooperation only accounting for 7.1% of the total research outputs, suggesting that China places more emphasis on domestic cooperation. Nonetheless, China continued to excel in international collaboration, working with ten countries, with the U.S. being its closest partner, with 17 collaborations (**Figure 3B**). The U.S. has cooperated with five countries, in particular with Canada on 14 occasions, and more than one-third of its publications were multinational and widely recognized internationally.

**Table 2** elucidates the correlation between the number of publications and citations among the top ten prolific countries. Intriguingly, China has emerged as the foremost contributor in terms of publications and total citations, but its average article citation rate fell considerably below that of the subsequent countries, the U.S. and South Korea. This indicates that China’s relevant publications exhibit an uneven quality and necessitate further attention. Canada has published only three articles, with an average of 53 citations, demonstrating its enormous international influence.

**Table 2:** Number of publications and citations by country.

Countries	Articles	Total Citations	Average Article Citations
China	266	2747	10.3
US	40	663	16.6
South Korea	24	397	16.5
Brazil	16	150	9.4
Australia	7	156	22.3
Sweden	7	145	20.7
UK	6	109	18.2
Japan	5	24	4.8
Canada	3	159	53.0
Germany	3	21	7.0

### 3.3 Institutional analysis

**Figure 4:**
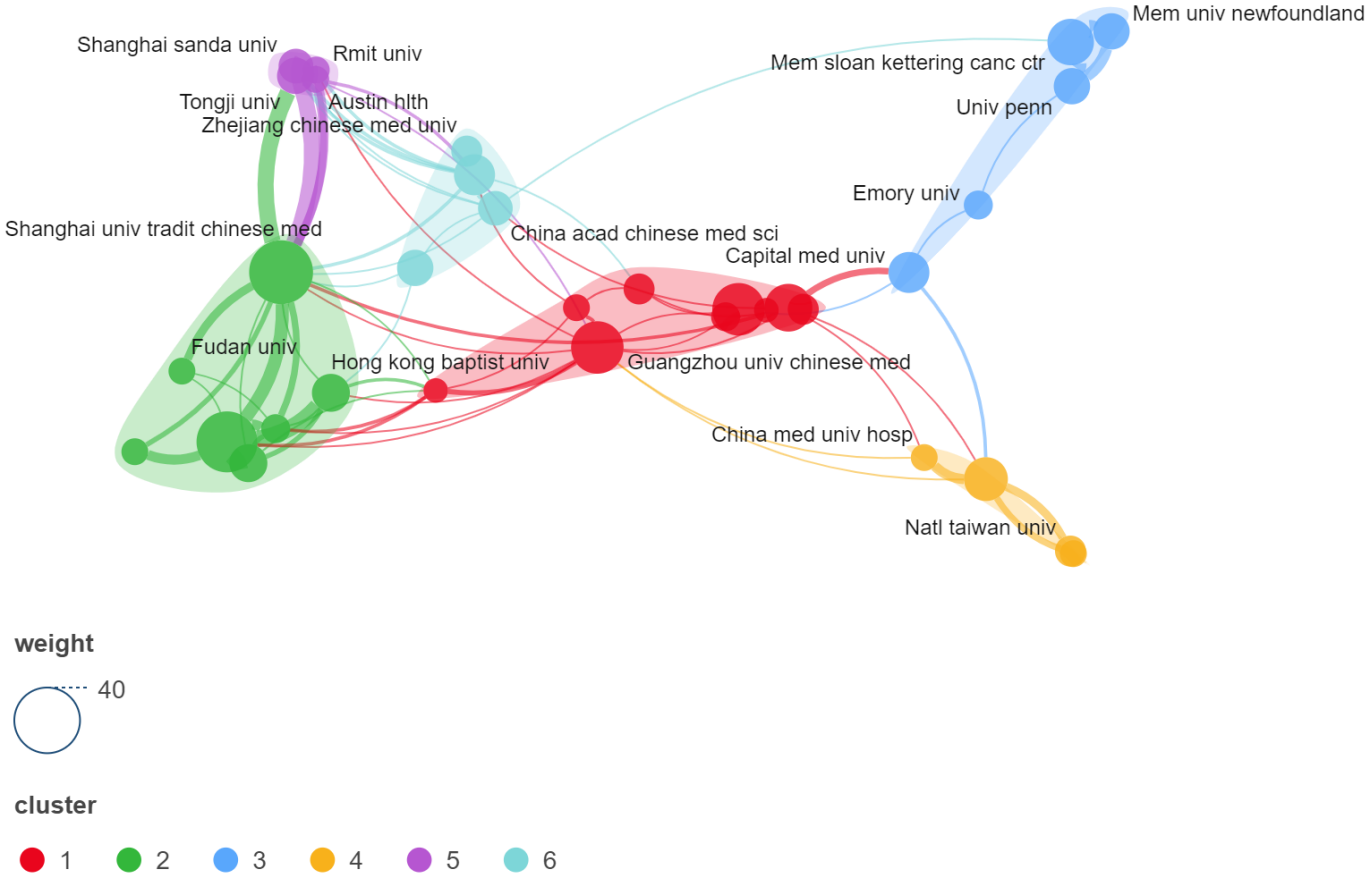
Institutional co-authorship analysis visualization network.

Between 2004 and 2023, a total of 465institutions globally published scholarly works related to acupuncture for sleep disorders. **Figure 4** illustrates a visual network for inter-institutional co-authorship analysis, with nodes representing institutions and the size of the nodes reflecting the number of publications. The lines represent cooperative relationships between institutions, and the thickness of the lines is proportional to the strength of the association. Institutions with at least five publications were included in the analysis, which contained six clusters and 33 nodes after removing isolated unconnected nodes. The University of Hong Kong (papers: 81; total link strength: 44), Shanghai University of Traditional Chinese Medicine (papers: 75; total link strength: 60), Memorial Sloan-Kettering Cancer Center (papers: 50; total link strength: 21), and Guangzhou University of Traditional Chinese Medicine (papers: 41; total link strength: 16) occupied the pivotal positions of the visualized network graph. Cluster 1 (red) consisted of nine institutions, dominated by the Guangzhou University of Traditional Chinese Medicine, Chengdu University of Traditional Chinese Medicine, and Beijing University of Traditional Chinese Medicine. The cluster’s sphere of influence extended to five provinces in China and linked to all four other clusters. Cluster 2 (green) was divided into two sub-clusters based on the proximity of the nodes: one dominated by the University of Hong Kong and the Hong Kong Baptist University, and the other consisting of institutions in Shanghai, such as the Shanghai University of Traditional Chinese Medicine and Fudan University. Cluster 3 (blue) consisted primarily of North American research institutions such as Emory University, Sloan-Kettering Cancer Institute, and the University of Pennsylvania. Cluster 4 (yellow) concentrated on early research institutions engaged in acupuncture for sleep disorders, such as China Medical University and National Taiwan University. Cluster 5 (purple) was an emerging research collective that included institutions such as RMIT University, Tongji University, and Shanghai Sanda College. Cluster 6 (ice) was small and consisted mainly of Chinese universities, led by Changchun Medical University. A clear geographical pattern can be seen in the cooperation of these institutions.

### 3.4 Sources analysis

**Figure 5:**
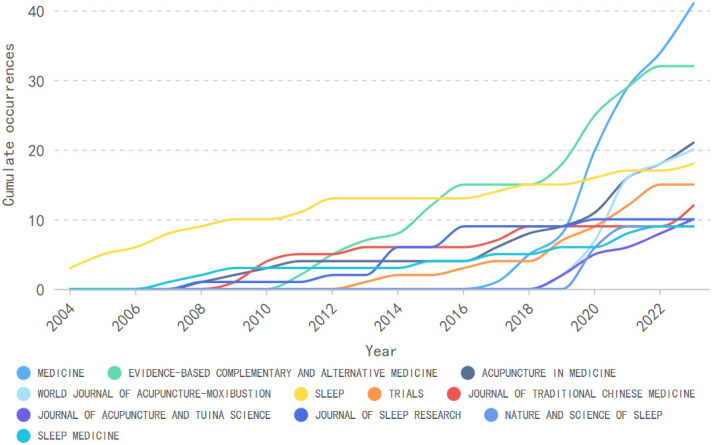
Trends of publications in the top 10 sources.

Over the past two decades, 146 sources have published articles on acupuncture for sleep disorders. According to **Table 3**, the top 11 sources were all journals, accounting for 45.83% (198/432) of the total publications. Analysis of the journal citation reports reveals that only 27.27% of these journals were classified as Q1 or Q2. Among them, 45.45% belonged to the field of complementary medicine, while 36.36% were associated with clinical neurology. The local citation score indicates the number of citations an article receives from other publications in the same field and can reflect the publication’s impact (29). MEDICINE was the most published journal in the field (9.72% of the total), but it received only 69 local citations. As shown in **Figure 5**, the journal experienced a significant surge in publications in this field starting in 2020, exceeding 40 in 2023. In contrast, SLEEP has established itself as a leading authority in the field, boasting the highest number of local citations, amounting to 651. The identification of such authoritative journals is helpful for researchers in acupuncture for sleep disorders to gain insight into current research trends and hotspots.

**Table 3:** Most relevant sources.

Ranking	Sources	Articles	Local Citations	JCR-c (IF)
1	MEDICINE	42	69	Q3 (1.60)
2	EVIDENCE-BASED COMPLEMENTARY AND ALTERNATIVE MEDICINE	32	306	Q3 (2.65) *
3	ACUPUNCTURE IN MEDICINE	21	193	Q3 (2.50)
4	WORLD JOURNAL OF ACUPUNCTURE - MOXIBUSTION	20	29	Q4 (0.70)
5	SLEEP	18	651	Q1 (5.60)
6	TRIALS	15	65	Q4 (2.50)
7	JOURNAL OF TRADITIONAL CHINESE MEDICINE	12	120	Q3 (2.60)
8	JOURNAL OF ACUPUNCTURE AND TUINA SCIENCE	10	33	Q4 (0.50)
9	JOURNAL OF SLEEP RESEARCH	10	107	Q2 (4.40)
10	NATURE AND SCIENCE OF SLEEP	9	55	Q3 (3.40)
11	SLEEP MEDICINE	9	412	Q2 (4.80)

JCR-c, Joumal Ctation Reports category (2022); IF, impact factor (2022); *The journal was removed from the SCI Catalog (2022) and adopted the 2021 edition.

### 3.5 Authors analysis

**Figure 6:**
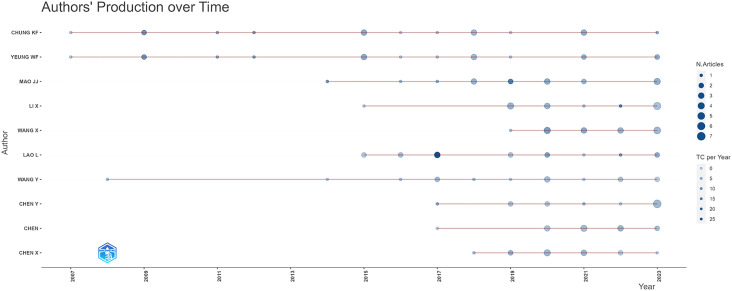
Authors production over time.

A total of 1,657 authors were associated with the 432 publications selected. **Figure 6** illustrates the productivity of the top 10 authors in the respective field throughout the years. Leading the list is Ka-Fai Chung from the Department of Psychiatry at the University of Hong Kong. He initiated his research on acupuncture for sleep disorders in 2007 and has contributed 18 papers on the subject, accumulating a total of 518 citations. Furthermore, the most influential article in the field, published in 2017, is a collaborative clinical study on the effectiveness and safety of acupuncture for primary insomnia by Shifen Xu from the Shanghai University of Traditional Chinese Medicine and Lixing Lao from the University of Hong Kong, with 129 total citations and 16.13 citations per year. **Figure 6** also demonstrates an increasing number of researchers exploring acupuncture as a potential treatment for sleep disorders within the past five years, suggesting a promising outlook for its application.

### 3.6 Analysis of research hotspots

#### 3.6.1 Most cited articles

Citation analysis helps to elucidate the interconnections and influence of publications within a specific research domain (30). **Table 4** presents the citation rankings for articles on acupuncture for sleep disorders. All but one systematic review and one meta-analysis were clinical studies, and 80% of the studies focused on insomnia. The publication titled “Acupuncture Increases Nocturnal Melatonin Secretion and Reduces Insomnia and Anxiety: A Preliminary Report” from 2004 garnered the highest number of citations, amounting to 138, thus presenting initial evidence regarding the efficacy of acupuncture in ameliorating sleep disorders, particularly anxiety-induced insomnia. The article “Acupuncture Versus Cognitive Behavioral Therapy for Insomnia in Cancer Survivors: A Randomized Clinical Trial” published in the Journal of the National Cancer Institute in 2019 was ranked 7th, a highly cited article in recent years. The article compared acupuncture with cognitive-behavioral therapy and demonstrated that both were effective in improving insomnia among cancer survivors. Acupuncture was found to be more suitable for concurrent pain management. These findings suggest that acupuncture has been increasingly advocated for the management of both primary and secondary sleep disorders.

**Table 4:** Top 10 articles in total citations.

Rank	Title	Total Citations	Annual citations	DOI
1	Acupuncture increases nocturnal melatonin secretion and reduces insomnia and anxiety: a preliminary report	138	6.57	10.1176/appi.neuropsych.16.1.19
2	Efficacy and safety of acupuncture treatment on primary insomnia: a randomized controlled trial	129	16.13	10.1016/j.sleep.2017.02.012
3	Acupuncture for treatment of insomnia: a systematic review of randomized controlled trials	107	6.69	10.1089/acm.2009.0041
4	Acupuncture for insomnia	106	8.15	10.1002/14651858.CD005472.pub3
5	Electroacupuncture for fatigue, sleep, and psychological distress in breast cancer patients with aromatase inhibitor-related arthralgia: a randomized trial	102	9.27	10.1002/cncr.28917
6	Electroacupuncture for primary insomnia: a randomized controlled trial	102	6.38	10.1093/sleep/32.8.1039
7	Acupuncture Versus Cognitive Behavioral Therapy for Insomnia in Cancer Survivors: A Randomized Clinical Trial	89	14.83	10.1093/jnci/djz050
8	Electroacupuncture for residual insomnia associated with major depressive disorder: a randomized controlled trial	88	6.29	10.5665/SLEEP.1056
9	Acupuncture and reflexology for insomnia: a feasibility study	86	5.38	10.1136/aim.2009.000760
10	Acupressure and Transcutaneous Electrical Acupoint Stimulation in improving fatigue, sleep quality and depression in hemodialysis patients	76	3.67	10.1142/S0192415X04002065

#### 3.6.2 Reference analysis for citation bursts

**Figure 7:**
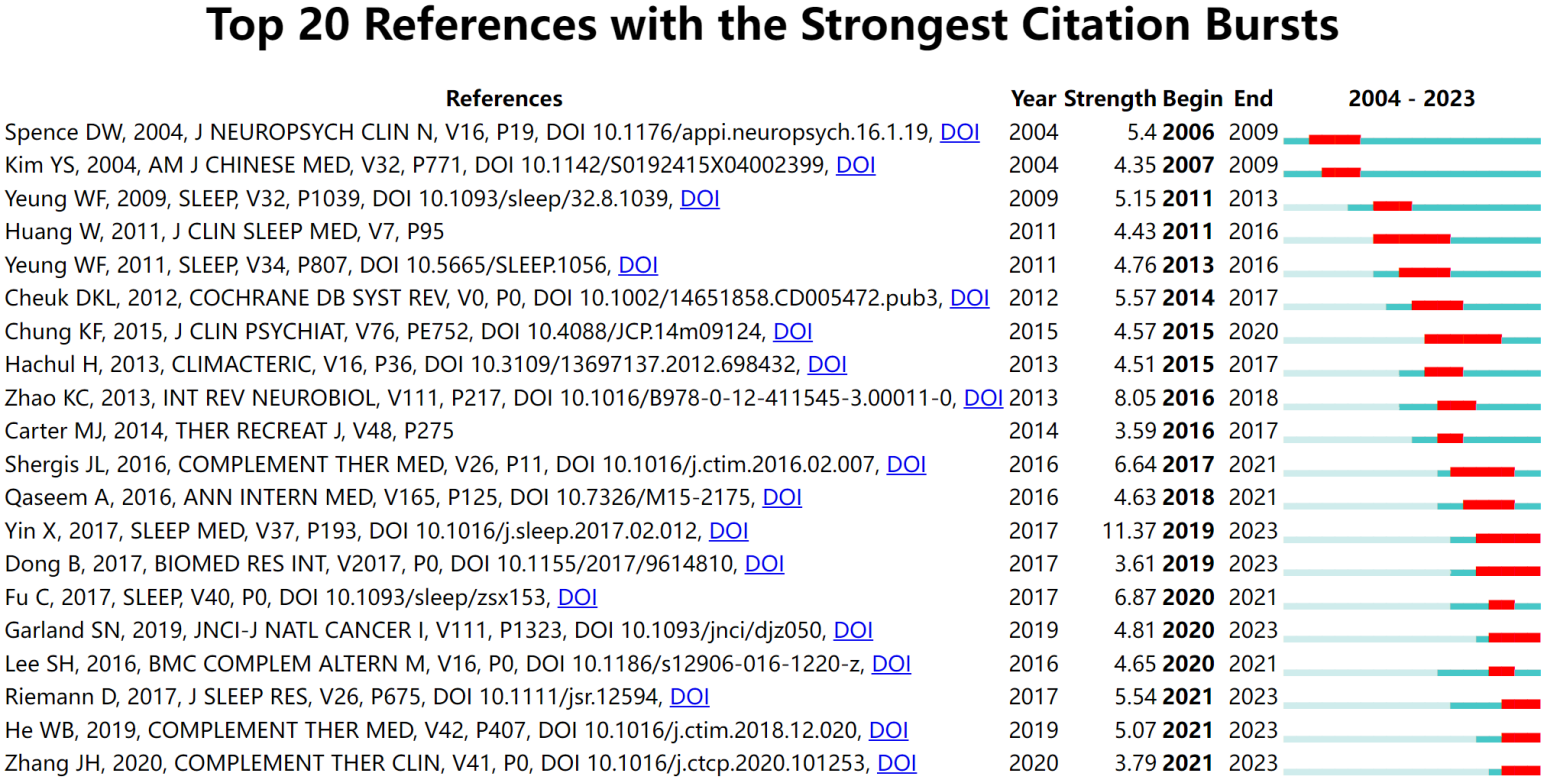
Top 19 publications with the highest citation bursts.

Citation burst refers to a surge in citations to a particular publication within a specific timeframe (31). **Figure 7** shows the 20 publications on acupuncture for sleep disorders that have experienced the highest citation bursts over the past two decades. In the figure, the light blue line represents the pre-publication period of the article, the dark blue line signifies the post-publication period, and the red line denotes the duration of the citation burst. For instance, the article “Efficacy and safety of acupuncture treatment on primary insomnia: a randomized controlled trial” published in 2017 attained the highest citation burst value of 11.37. Furthermore, the ongoing citation bursts in six articles, such as “The Efficacy of Acupuncture for Treating Depression-Related Insomnia Compared with a Control Group: A Systematic Review and Meta-Analysis” suggests that research on acupuncture for secondary insomnia remains prevalent.

#### 3.6.3 Analysis of the most frequently used keywords

**Figure 8:**
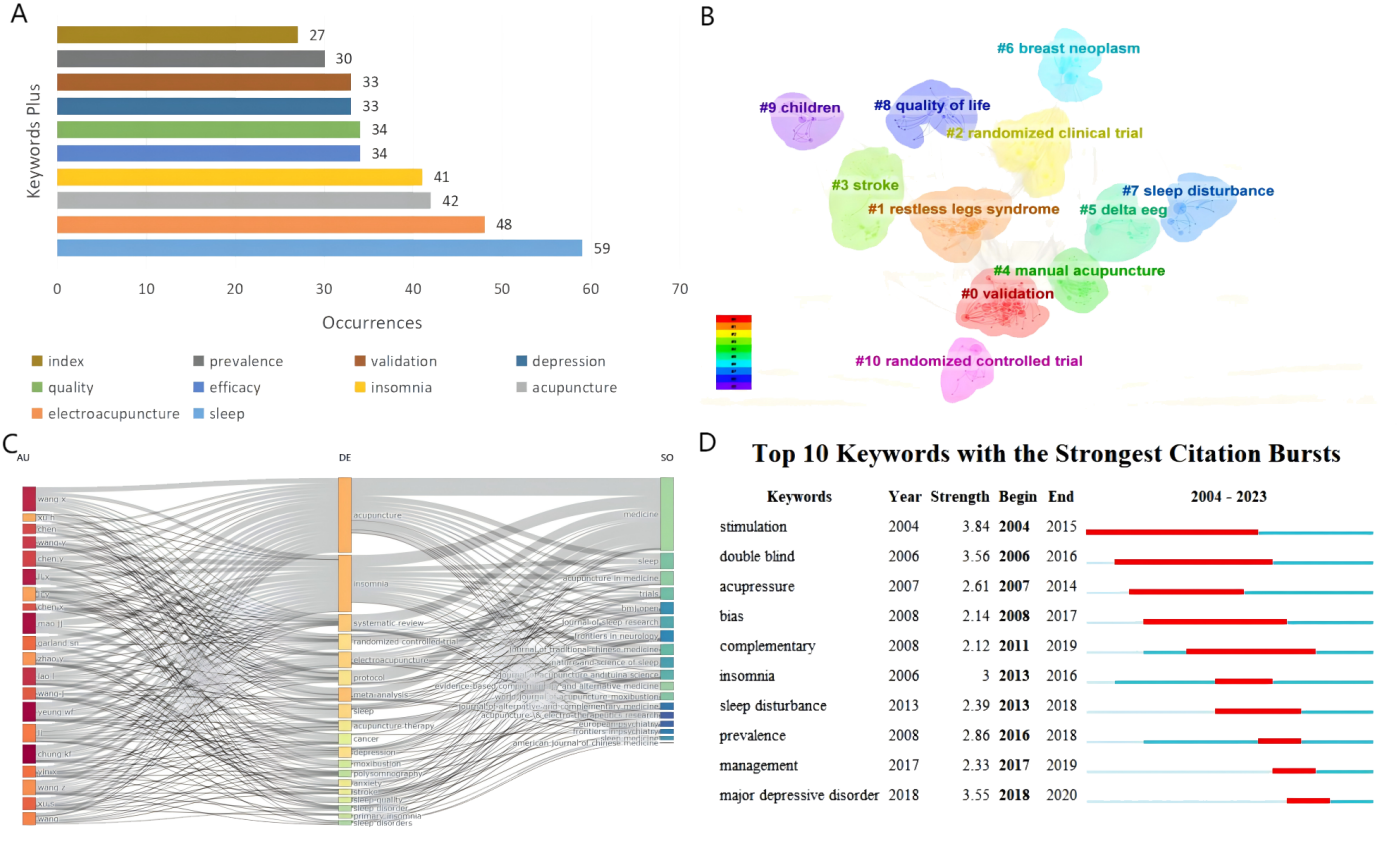
**(A)** Top 10 most frequent keywords; **(B)** Clustering of keywords; **(C)** Three-factor map of authors, keywords, and sources; **(D)** Top 10 keywords with the strongest citation bursts.

**Figure 8A** illustrates the ten most commonly used keywords and their frequency in studies on acupuncture for sleep disorders. “Sleep,” “electroacupuncture,” and “acupuncture” ranked in the top 3, representing the major research fields. “Insomnia” was the dominant category of acupuncture for sleep disorders. Additionally, keywords like “quality,” “validation,” and “efficacy” imply that the effectiveness of acupuncture for sleep disorders remains a hot topic at present.

Keyword clustering relies on co-occurrence analysis and employs statistical techniques to condense the co-occurrence network into a limited number of clusters, wherein keywords sharing common themes are grouped (32). In general, silhouette values above 0.7 provide reliable clustering results (33). **Figure 8B** visually presents the keyword clustering of acupuncture for sleep disorders. The lower the label number, the greater the number of keywords encompassed within the clustering. The modularity value for this graph is 0.5048, while the silhouette value is 0.7845, indicating a well-formed cluster. The 644 keywords within the specified domains were divided into 11 clusters, specifically labeled as “validation,” “restless legs syndrome,” “randomized clinical trial,” “stroke,” “manual acupuncture,” “delta eeg,” “breast neoplasm,” “sleep disturbance,” “quality of life,” “children,” and “randomized controlled trial.”

**Figure 8C** shows a three-field plot depicting the authors, keywords, and sources with the highest relevance within the domain. The height of each rectangle in the plot is proportional to the relevance. It is evident that the most locally cited journal in this field, SLEEP, and the top-ranked author, Ka-Fai Chung, focus on the topics of “insomnia,” “electroacupuncture,” “randomized controlled trial,” and “depression”.

#### 3.6.4 Analysis of keywords citation bursts

**Figure 8D** lists the top 10 keywords with the highest citation bursts. The temporal sensitivity of the keywords has resulted in a noticeable absence of significant burst intensity depicted in this graph. However, certain keywords, such as “stimulation” (2004-2015), “double blind” (2006-2016), and “acupressure” (2007-2014) have consistently attracted attention over an extended period. In contrast, “major depressive disorder” (2018-2020), “management” (2017-2019), and “prevalence” (2016-2018) have experienced a surge in attention over the past five years. This trend indicates that the epidemiology, presentation, and management of sleep disorders are expected to remain prominent research topics in the field of acupuncture for sleep disorders.

## 4 Discussion

### 4.1 Global trends in acupuncture for sleep disorders

This study quantitatively analyzed the historical and present state of the development of acupuncture for sleep disorders over the past two decades. Within the last five years, there has been a notable increase in publications within this field compared to the preceding period. However, the average number of citations per year and per article has exhibited a contrasting trend, indicating a deficiency in high-quality and innovative publications regarding acupuncture for sleep disorders. A study of acupuncture for insomnia from 1999-2018 also confirmed a slow growth of pertinent literature until 2018 (22). Twenty-seven countries in Asia, Europe, North America, and Oceania have participated in studies of acupuncture for sleep disorders. China emerged as the primary contributor, but more than 90% of these contributions resulted from domestic collaborations, with an average article citation rate of 10.3, indicating a lack of significant international impact. The University of Hong Kong, Shanghai University of Traditional Chinese Medicine, and Guangzhou University of Chinese Medicine were the major research institutes. In contrast, the U.S. engaged in several high-quality international collaborations, particularly with Canada and South Korea, resulting in an average article citation rate of 16.6. This achievement can be attributed to the contributions made by various institutions such as the University of Pennsylvania, Stanford University, and the Memorial Sloan Kettering Cancer Center. Furthermore, the U.S. government has provided more healthcare support, with a per capita healthcare expenditure of $10,784 in 2021 alone, which may contribute to the abundance of noteworthy medical outcomes observed in the U.S. (34, 35). The primary influential sources within this field consist of scholarly journals that concentrate on complementary medicine and clinical neurology. Among these publications, MEDICINE emerged as the most active journal on acupuncture for sleep disorders. Additionally, the esteemed journal SLEEP has published high-level clinical randomized controlled trials of acupuncture for the treatment of perimenopausal insomnia, primary insomnia, and residual insomnia associated with major depression disorder (36–38). The quantification of publications can reflect an author’s engagement and impact within a specific scientific domain. Ka-Fai Chung from the University of Hong Kong has been the most prolific researcher, working on acupuncture for sleep disorders since 2007, with 18 related publications. In 2017, Shifen Xu from the Shanghai University of Traditional Chinese Medicine and Lixing Lao from the University of Hong Kong jointly conducted a clinical study to investigate the effectiveness and safety of acupuncture for primary insomnia. This study has been cited more than a hundred times to date (39).

### 4.2 Hot and emerging frontiers in acupuncture for sleep disorders

Citation analysis, citation bursting, keyword analysis, and keyword bursting are widely employed bibliometric techniques that offer insights into the evolution of research within a particular discipline and enable the anticipation of future research trends and focal points (40). Based on the available data, investigations about acupuncture for sleep disorders have primarily concentrated on five key domains: secondary insomnia, restless leg syndrome, electroacupuncture, effectiveness, and mechanisms.

#### 4.2.1 Secondary insomnia

Insomnia, characterized by frequent difficulties in initiating and maintaining sleep, and dissatisfaction with the sleep quality, is the most common sleep disorder (41). More than 30% of the global adult population has experienced insomnia, and approximately 40% of these individuals have developed chronic insomnia disorder (≥3 months) (41, 42). Among these cases, insomnia cases caused by somatic diseases, mental disorders, substance abuse, and environmental changes are referred to as “secondary insomnia” or “co-morbid insomnia” (43). It is sometimes difficult to establish a causal relationship between these disorders and insomnia, such as chronic pain syndromes and depression, making the diagnosis and treatment of secondary insomnia difficult (44, 45). **Table 4** shows that the top 10 most cited articles focused on the clinical effects of acupuncture for insomnia. When considering the findings from citation bursts, it becomes apparent that recent research has increasingly explored the role of acupuncture in addressing insomnia associated with conditions such as cancer, stroke, and perimenopause. This underscores acupuncture’s ongoing potential as an alternative therapeutic option (36, 46, 47).

#### 4.2.2 Restless legs syndrome

Restless legs syndrome, a prevalent sleep-related movement disorder, manifests as intense discomfort in the legs at rest, characterized by sensations of tingling, burning, and tightness, with a prevalence of 18%-23% in older adults (48). This disorder may arise either spontaneously or as a consequence of iron deficiency anemia, pregnancy, diabetes, or other contributing factors (49). Dopamine agonists, namely pramipexole, ropinirole, and rotigotine, have received approval from both the U.S. Food and Drug Administration and the European Medicines Agency as primary treatment options for restless legs syndrome. However, these medications still exhibit varying levels of adverse effects and potentiation (50). In recent years, non-pharmacological interventions such as exercise, acupuncture, and repetitive transcranial magnetic stimulation have gained recognition for their potential role in managing this condition. Several clinical randomized controlled trials have provided evidence that acupuncture effectively alleviates leg discomfort and reduces nocturnal activity in restless leg syndrome without causing side effects (14, 51, 52). In the context of keyword clustering, the keywords represented by restless leg syndrome were the second largest cluster, indicating the progressive use of acupuncture for diverse sleep disorders beyond insomnia.

#### 4.2.3 Electroacupuncture

Acupuncture encompasses a range of techniques, such as manual acupuncture, electroacupuncture, warming needle moxibustion, auricular acupuncture, and acupoint injections (53). According to the citation bursts of keywords, “acupressure” has received constant attention over an extended period of time (2007-2014). Acupressure, a non-invasive therapeutic method, is used to achieve therapeutic effects by applying pressure to specific acupoints on the body to regulate qi and blood in the meridians (54). Due to its simplicity in acquisition, acupressure frequently serves as an initial therapeutic approach for sleep disorders, aiming to circumvent the potential detrimental consequences associated with pharmacological interventions. As the global recognition of acupuncture has grown, the prevailing method for addressing sleep disorders has shifted from acupressure to electroacupuncture, as evidenced by the frequency of keyword occurrences. Electroacupuncture is a technique in which low-frequency pulsed currents close to the bioelectricity of the human body are applied to the needles to prevent and treat diseases after the needles are inserted into the acupoints and produce a “deqi” sensation (55). According to previous studies, electroacupuncture can be used to treat various sleep disorders, including primary and secondary insomnia, circadian rhythm sleep-wake disorders, and restless legs syndrome, by manipulating the frequency, intensity, and acupoints (14, 56, 57).

#### 4.2.4 Effectiveness

While cognitive-behavioral therapy is the primary non-pharmacological treatment option acknowledged for sleep disorders, its clinical prevalence is limited due to the need for doctor-patient collaboration and the time-intensive nature of the therapy (58). Consequently, as a complementary alternative therapy, acupuncture has gained considerable usage in the treatment of sleep disorders. However, it remains unclear whether existing evidence is sufficient to support acupuncture for all types of sleep disorders. In the context of insomnia treatment through acupuncture, multiple meta-analyses have demonstrated that the existing evidence is inadequate to establish or dismiss the efficacy of acupuncture for insomnia (19, 59). This insufficiency arises due to the susceptibility of relevant clinical trials to publication bias, owing to the considerable discrepancies in defining insomnia, participant attributes, and study methodologies. “Quality,” “validation,” and “efficacy” were among the top 10 keywords, confirming that clinical trials of acupuncture for sleep disorders are still not standardized. Furthermore, “validation” represented the primary major cluster in the keyword clustering, suggesting that the effectiveness and safety of acupuncture for sleep disorders are still under continuous scrutiny.

#### 4.2.5 Mechanisms

Clinical evidence indicates that acupuncture has been effective in enhancing both the quality and duration of sleep. However, the precise mechanism underlying this effect remains unclear (56). Drawing on previous research, we present a summary of the potential mechanisms through which acupuncture may alleviate sleep disorders. (1) The principle of warming needle moxibustion to improve insomnia is directly related to gene regulation. This acupuncture technique involves inserting moxa sticks into the handles of acupuncture needles after needling the acupoints to dredge collaterals and strengthen the immune system through warm stimulation. A study found that warming needle moxibustion enhanced the expression of microRNA-101a and suppressed the expression of paired-box 8 in the hippocampus of rat models with insomnia (60). In addition, warming needle moxibustion upregulated the transcript levels of brain-derived neurotrophic factor (BDNF), recombinant early growth response protein 1, and b-cell translocation gene 2, protecting the brain from insomnia-related damage (61). (2) The gut microbiota is closely related to sleep, and acupuncture may treat insomnia by modulating host immune responses through gut flora such as Lactobacillus (62, 63). (3) The hypothalamic-pituitary-adrenal axis plays a crucial role in regulating the stress response and serves as a significant component of the neuroendocrine system. Research has demonstrated that electroacupuncture enhances the expression of D1 and D2 receptors in the hypothalamus, reduces the levels of corticotropin-releasing hormone, adrenocorticotropic hormone, and cortisol, mitigates stress-induced changes in neurotransmitters, and ameliorates acute stress-induced insomnia (64). (4) Patients with chronic insomnia exhibit neuronal cell damage in the cerebral cortex, including degeneration, denaturation, and apoptosis. Research has demonstrated that electroacupuncture can modulate apoptosis in insomniac rats by increasing the expression of B-cell lymphoma-2, reducing the expression of Bax, Bcl-xL/Bcl-2-associated death promoter, and caspase-3. This therapeutic approach also targets the PI3K/AKT pathway and the CAMP/CREB pathway to regulate apoptosis and exerts a beneficial effect on monoamine neurotransmitters (65). Furthermore, proteomics data indicate that acupuncture improves sleep by modulating the expression of four neural-related proteins, Prolargin, NMDA receptor synaptonuclear-signaling and neuronal migration factor, Transmembrane protein 41B, and Microtubule-associated protein 1B (66). (5) Electroacupuncture has been shown to enhance memory deficits resulting from sleep deprivation. This effect is attributed to the activation of the BDNF/TrkB/Erk signaling pathway, which promotes the survival of hippocampal neurons and facilitates synaptic plasticity. Furthermore, electroacupuncture has the potential to partially restore dopamine levels in the hippocampus by activating calcium/calmodulin protein-dependent protein kinase II, synaptophysin I, and tyrosine hydroxylase (16, 67). (6) For secondary sleep disorders, 10-Hz electroacupuncture stimulation of the Fengchi (GB20) has been found to suppress focal epilepsy and its associated sleep disorders. This suppression may be related to the involvement of opioid receptors in the central nucleus of the amygdala (68). Shenmen (HT7) is the most commonly used acupoint for sleep disorders and has been widely used to treat neuropsychological disorders such as amnesia, epilepsy, and insomnia (69). Acupuncture at HT7 alleviated caffeine-dependent sleep deprivation by modulating neuronal activity in the basal forebrain arousal region and BDNF-mediated endoplasmic reticulum stress in the medial septum (17, 70). In summary, acupuncture improves sleep disorders by orchestrating a multifaceted mechanism involving various pathways and targets, encompassing the nervous system, endocrine system, and intestinal flora.

## 5 Limitations

This study is subject to several limitations. Firstly, the data were exclusively obtained from WoSCC, which, while encompassing a substantial portion of high-quality publications in the field, may still have overlooked certain sources. Secondly, due to the complexity of sleep disorder classification and to avoid the inclusion of irrelevant literature, we used a title search but may have missed a few articles. Thirdly, the names of institutions or sources may have changed over time, given the temporal nature of the study. Lastly, it is important to note that academic publications often lag behind clinical reality, thus the findings of this study solely reflect the current research trends in academia, which was the initial purpose of this bibliometric analysis.

## 6 Conclusion

The study conducted a bibliometric analysis of 432publications in the WoSCC on acupuncture for sleep disorders from 2004 to 2023. As acupuncture techniques continue to gain global recognition, more and more scholars are dedicating themselves to the field. In addition to insomnia, acupuncture is increasingly being used to treat a variety of sleep disorders, with electroacupuncture becoming the new superior technique. However, the effectiveness, safety, and practicability of acupuncture for sleep disorders remain uncertain due to the inadequate quality of prior research. Acupuncture can modulate various targets and pathways within the organism, yet the precise underlying mechanism necessitates further investigation. Nonetheless, we believe that developing standardized protocols for subsequent clinical trials and exploring the mechanisms of acupuncture for sleep disorders will contribute to a broader understanding of the field.

## Data Availability

The original contributions presented in the study are included in the article/**Supplementary Material**. Further inquiries can be directed to the corresponding authors.
