# Supplementary material for: Current perspectives and trends in acupuncture for sleep disorders: a bibliometric analysis
Source: Front Psychiatry. 2024 Oct 29;15:1338455. doi: 10.3389/fpsyt.2024.1338455 (PMC11554521; doi:10.3389/fpsyt.2024.1338455)
Supplement: Supplementary file 1 [file DataSheet1.pdf]

Web of Science Search Strategy (v0.1)

Database: Web of Science Core Collection

Entitlements:

- WOS.IC: 1993 to 2024
- WOS.CCR: 1985 to 2024
- WOS.SCI: 1900 to 2024
- WOS.AHCI: 1998 to 2024
- WOS.ESCI: 2019 to 2024
- WOS.ISTP: 1998 to 2024
- WOS.SSCI: 1998 to 2024
- WOS.ISSHP: 1998 to 2024

Searches:

1: (“acupuncture” OR “needling” OR “electroacupuncture” OR “electro-acupuncture” OR “warm needling” OR “warm acupuncture” OR “fire needle” OR “dry needling” OR “scalp acupuncture” OR “body acupuncture” OR “ear acupuncture” OR “auricular acupuncture” OR “abdominal acupuncture” OR “wrist-ankle acupuncture” OR “manual acupuncture” OR “Moxibustion” OR “acupoint” OR “acupoint injection”) (Title) AND (“sleep*” OR “insomnia” OR “sleep-related” OR “hypoventilation” OR “hypersomn*” OR “narcolepsy” OR “Kleine-Levin syndrome” OR “sleep-wake” OR “jet lag” OR “nightmare” OR “Parasomnia*” OR “confusional arousals” OR “exploding head syndrome” OR “restless legs syndrome” OR “periodic limb movement disorder”) (Title) Date Run: Sun Oct 06 2024 13:17:26 GMT+0800

2: (“acupuncture” OR “needling” OR “electroacupuncture” OR “electro-acupuncture” OR “warm needling” OR “warm acupuncture” OR “fire needle” OR “dry needling” OR “scalp acupuncture” OR “body acupuncture” OR “ear acupuncture” OR “auricular acupuncture” OR “abdominal acupuncture” OR “wrist-ankle acupuncture” OR “manual acupuncture” OR “Moxibustion” OR “acupoint” OR “acupoint injection”) (Title) AND (“sleep*” OR “insomnia” OR “sleep-related” OR “hypoventilation” OR “hypersomn*” OR “narcolepsy” OR “Kleine-Levin syndrome” OR “sleep-wake” OR “jet lag” OR “nightmare” OR “Parasomnia*” OR “confusional arousals” OR “exploding head syndrome” OR “restless legs syndrome” OR “periodic limb movement disorder”) (Title) and 2023 or 2022 or 2021 or 2020 or 2019 or 2018 or 2017 or 2016 or 2015 or 2014 or 2013 or 2012 or 2011 or 2010 or 2009 or 2008 or 2007 or 2006 or 2005 or 2004 (Publication Years) Date Run: Sun Oct 06 2024 13:17:50 GMT+0800

3: (“acupuncture” OR “needling” OR “electroacupuncture” OR “electro-acupuncture” OR “warm needling” OR “warm acupuncture” OR “fire needle” OR “dry needling” OR “scalp acupuncture” OR “body acupuncture” OR “ear acupuncture” OR “auricular acupuncture” OR “abdominal acupuncture” OR “wrist-ankle acupuncture” OR “manual acupuncture” OR “Moxibustion” OR “acupoint” OR “acupoint injection”) (Title) AND (“sleep*” OR “insomnia” OR “sleep-related” OR “hypoventilation” OR “hypersomn*” OR “narcolepsy” OR “Kleine-Levin syndrome” OR “sleep-wake” OR “jet lag” OR “nightmare” OR “Parasomnia*” OR “confusional arousals” OR “exploding head syndrome” OR “restless legs syndrome” OR “periodic limb movement disorder”) (Title) and 2023 or 2022 or 2021 or 2020 or 2019 or 2018 or 2017 or 2016 or 2015 or 2014 or 2013 or 2012 or 2011 or 2010 or 2009 or 2008 or 2007 or 2006 or 2005 or 2004 (Publication Years) and English (Languages) Date Run: Sun Oct 06 2024 13:18:13 GMT+0800

(N-Výh QÆeö•ô) R e s u l t s : 4 3 6

Four papers published in 2024 were removed after manual screening Results: 432
